# Sodium dirubidium citrate, NaRb_2_C_6_H_5_O_7_, and sodium dirubidium citrate dihydrate, NaRb_2_C_6_H_5_O_7_(H_2_O)_2_

**DOI:** 10.1107/S2056989019003190

**Published:** 2019-03-11

**Authors:** Andrew J. Cigler, James A. Kaduk

**Affiliations:** a Department of Chemistry, North Central College, 131 S. Loomis St, Naperville, IL 60540, USA

**Keywords:** powder diffraction, density functional theory, citrate, sodium, rubidium, crystal structure

## Abstract

The crystal structures of sodium dirubidium citrate {poly[μ-citrato-dirubi­dium(I)sodium(I)], [NaRb_2_(C_6_H_5_O_7_)]_*n*_} and sodium dirubidium citrate dihydrate {poly[di­aqua­(μ-citrato)dirubidium(I)sodium(I)], [NaRb_2_(C_6_H_5_O_7_)(H_2_O)_2_]_*n*_} have been solved and refined using laboratory X-ray powder diffraction data, and optimized using density functional techniques. Both structures contain Na chains and Rb layers, which link to form different three-dimensional frameworks. In each structure, the citrate triply chelates to the Na^+^ cation. Each citrate also chelates to Rb^+^ cations. In the dihydrate structure, the water mol­ecules are bonded to the Rb^+^ cations; the Na^+^ cation is coordinated only to citrate O atoms. Both structures contain an intra­molecular O—H⋯O hydrogen bond between the hy­droxy group and one of the terminal carboxyl­ate groups. In the structure of the dihydrate, each hydrogen atom of the water mol­ecules participates in a hydrogen bond to an ionized carboxyl­ate group.

## Abstract 2

The crystal structures of sodium dirubidium citrate and sodium dirubidium citrate dihydrate have been solved and refined using laboratory X-ray powder diffraction data, and optimized using density functional techniques. Both structures contain Na chains and Rb layers, which link to form different three-dimensional frameworks.

## Chemical context   

**Figure 1:**
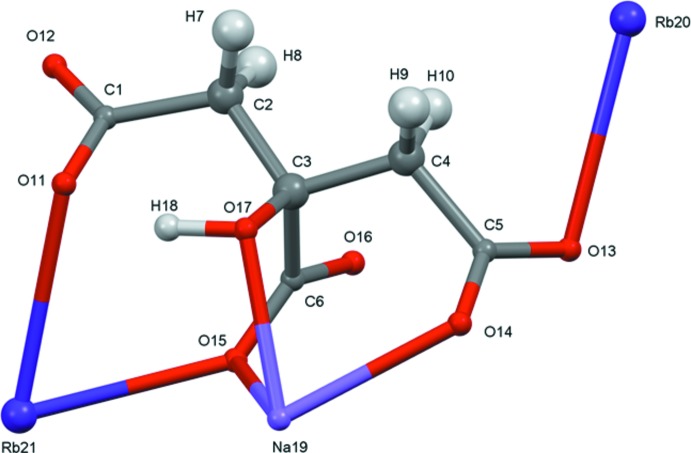
The asymmetric unit of NaRb_2_C_6_H_5_O_7_, with the atom numbering and 50% probability spheroids.

A systematic study of the crystal structures of Group 1 (alkali metal) citrate salts has been reported in Rammohan & Kaduk (2018 ▸). The study was extended to lithium metal hydrogen citrates in Cigler & Kaduk (2018 ▸), and to sodium metal hydrogen citrates in Cigler & Kaduk (2019 ▸). These two compounds (Figs. 1 ▸ and 2 ▸) are a further extension to sodium dirubidium citrates.
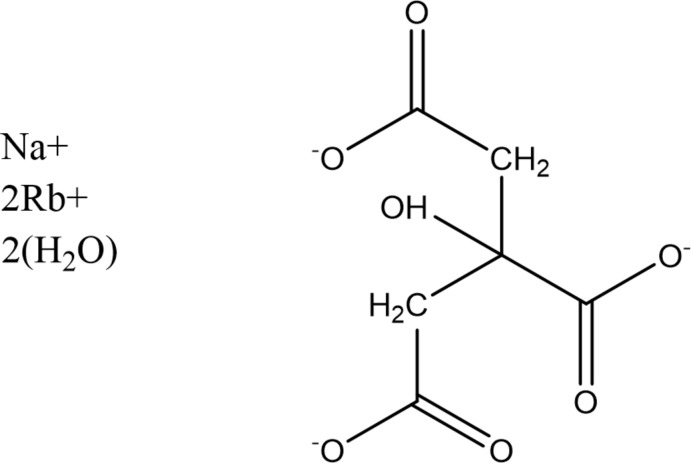


## Structural commentary   

**Figure 3:**
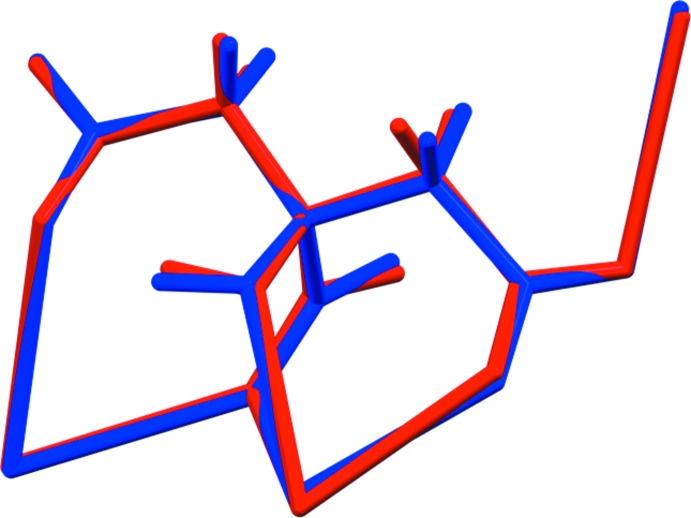
Comparison of the refined and optimized structures of sodium dirubidium citrate. The refined structure is in red, and the DFT-optimized structure is in blue.

**Figure 4:**
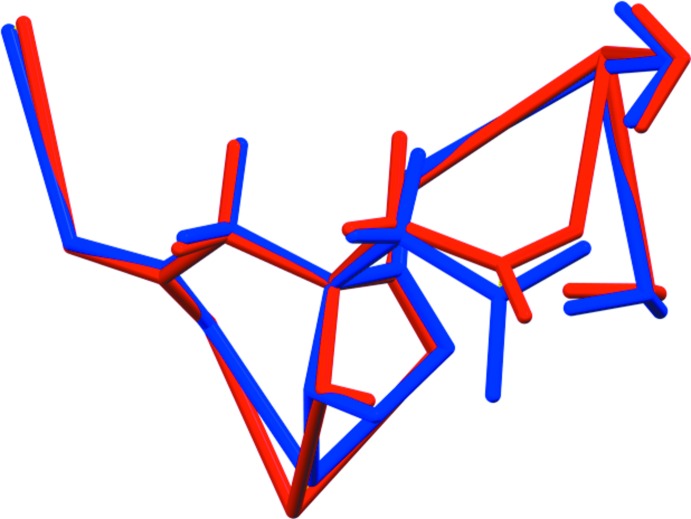
Comparison of the refined and optimized structures of sodium dirubidium citrate dihydrate. The refined structure is in red, and the DFT-optimized structure is in blue.

For NaRb_2_C_6_H_5_O_7_, the root-mean-square deviation of the non-hydrogen atoms in the refined and optimized structures is 0.095 Å (Fig. 3 ▸). The excellent agreement between the structures is strong evidence that the experimental structure is correct (van de Streek & Neumann, 2014 ▸). For NaRb_2_C_6_H_5_O_7_(H_2_O)_2_, the agreement of the refined and optimized structures is poorer (Fig. 4 ▸); the r.m.s. cartesian displacement is 0.45 Å. The largest differences are in the carboxyl group C5/O13/O14. Removing O13 and O14 from the displacement calculation yields a value of 0.222 Å, in the upper range of correct structures according to van de Streek & Neumann (2014 ▸). Apparently the refined structure is in error, perhaps because it was refined using laboratory X-ray powder data and the structure contains two heavy Rb atoms. This discussion uses the DFT-optimized structures.

In both structures, all of the citrate bond lengths, bond angles, and torsion angles fall within the normal ranges indicated by a *Mercury* Mogul Geometry Check (Macrae *et al.*, 2008 ▸). The citrate anion in both structures occurs in the *trans,trans*-conformation (about C2—C3 and C3—C4), which is one of the two low-energy conformations of an isolated citrate (Rammohan & Kaduk, 2018 ▸). The central carboxyl­ate group and the hy­droxy group exhibit small twists (O15—C6—C3—O17 torsion angles of −16.0 and −18.2°) from the normal planar arrangement.

In NaRb_2_C_6_H_5_O_7_, the citrate anion triply chelates to Na19 through the terminal carboxyl­ate O14, the central carboxyl­ate O15, and the hydroxyl group O17. The citrate also chelates to Rb21 through the terminal carboxyl­ate O11 and the central carboxyl­ate O15. Each citrate oxygen atom bridges multiple metal atoms. The Na^+^ cation is six-coordinate, with a bond-valence sum of 1.12. The two Rb^+^ cations are seven-coordinate, with bond-valence sums of 0.99 and 1.16.

In the dihydrate, the citrate anion similarly triply chelates to Na19 through the terminal carboxyl­ate O12, the central carboxyl­ate O15, and the hy­droxy group O17 (the numberings of the oxygen atoms are partially arbitrary). Each terminal carboxyl­ate group chelates to a different Rb21 cation. Most of the oxygen atoms bridge multiple metal atoms, but O13 and O14 bind only to Rb cations, and O17 binds only to the Na^+^ cation. The Na coordination sphere is composed only of citrate oxygen atoms. Rb20 is coordinated by four H_2_O, and Rb21 is bonded to two H_2_O mol­ecules. Each water mol­ecule is coordinated to two Rb20 and and one Rb21 cations. The Na^+^ cation is six-coordinate (distorted octa­hedral), with a bond-valence sum of 1.19. The Rb20 and Rb21 cations are eight- and nine-coordinate, respectively. The coordination polyhedra are irregular, and the bond-valence sums are 0.94 and 1.03. The Mulliken overlap populations in both structures indicate that the Rb—O bonds are ionic, but that the Na—O bonds have some covalent character.

## Supra­molecular features   

**Figure 5:**
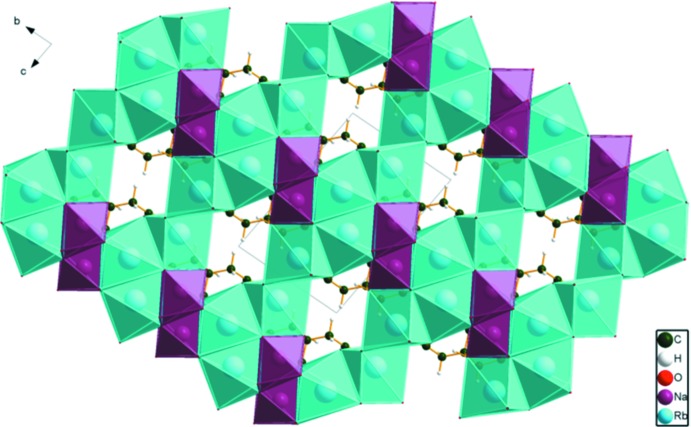
Crystal structure of NaRb_2_C_6_H_5_O_7_, viewed down the *a* axis.

In the crystal structure of NaRb_2_C_6_H_5_O_7_ (Fig. 5 ▸), the distorted octa­hedral NaO6 coordination polyhedra share edges to form zigzag double chains along the *a*-axis direction. The RbO_7_ polyhedra share edges to form layers parallel to the *ac* plane. These layers link the Na chains, forming a three-dimensional framework. The hydro­phobic methyl­ene groups of the citrate anions occupy cavities in this framework.

**Figure 6:**
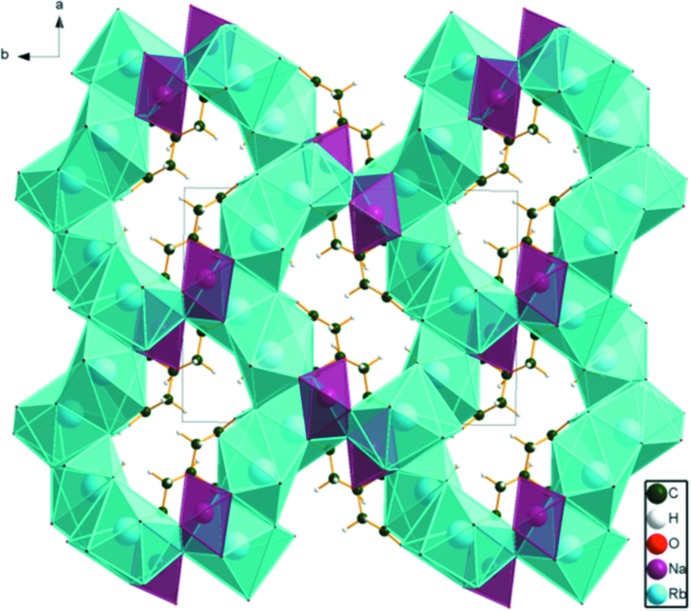
Crystal structure of NaRb_2_C_6_H_5_O_7_(H_2_O)_2_, viewed down the *a* axis.

In the crystal structure of NaRb_2_C_6_H_5_O_7_(H_2_O)_2_ (Fig. 6 ▸), the NaO_6_ coordination polyhedra share corners to form double zigzag chains along the *c*-axis direction. The Rb polyhedra share edges to form layers parallel to the *ac* plane. These layers share corners with each other and share edges with the Na chains, forming a three-dimensional framework. The hydro­phobic methyl­ene groups of the citrate anions also occupy cavities in this framework.

In NaRb_2_C_6_H_5_O_7_, the only traditional hydrogen bond is an intra­molecular O17—H18⋯O11 one between the hydroxyl group and one of the terminal carboxyl­ate groups (Table 1 ▸). By the correlation of Rammohan & Kaduk (2018 ▸), this hydrogen bond contributes 14.0 kcal mol^−1^ to the crystal energy. A weak C—H⋯O hydrogen bond also contributes to the crystal energy.

In NaRb_2_C_6_H_5_O_7_(H_2_O)_2_, each water mol­ecule hydrogen atom acts as a donor in an O—H⋯O hydrogen bond to a carboxyl­ate oxygen (Table 2 ▸). By the correlation of Rammohan & Kaduk (2018 ▸), these hydrogen bonds range from 11.0–14.0 kcal mol^−1^ in energy. There is an intra­molecular O17—H18⋯O13 hydrogen bond between the hydroxyl group and one of the terminal carboxyl­ate groups, as well as a C—H⋯O hydrogen bond.

**Figure 7:**
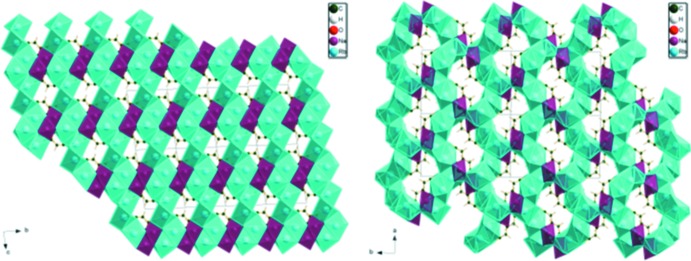
Comparison of the crystal structures of sodium dirubidium citrate (left) and sodium dirbuidium citrate dihydrate (right).

The two structures exhibit some similarities (Fig. 7 ▸), but a mechanism for inter­conversion of the structures is not obvious by visual inspection.

## Database survey   

Details of the comprehensive literature search for citrate structures are presented in Rammohan & Kaduk (2018 ▸). A reduced cell search for NaRb_2_HC_6_H_5_O_7_ in the Cambridge Structural Database (Groom *et al.*, 2016 ▸) yielded no hits, while that for NaRb_2_C_6_H_5_O_7_(H_2_O)_2_ yielded 21 hits, but when including the chemistry of C, H, Na, O, and Rb only it yielded no hits.

## Synthesis and crystallization   

NaRb_2_C_6_H_5_O_7_(H_2_O)_2_ was prepared by adding stoichiometric qu­anti­ties of Na_2_CO_3_ and Rb_2_CO_3_ to a solution of 10 mmol H_3_C_6_H_5_O_7_ in 10 ml of water. After the fizzing subsided, the clear solution was dried overnight at 348 K to yield a glass. This glass was heated at 450 K for 30 min to yield a pale-yellow solid. This solid was equilibrated in air at ambient conditions for 3 h. The anhydrous salt was prepared by heating the dihydrate at 450 K for 30 min.

## Refinement   

**Figure 8:**
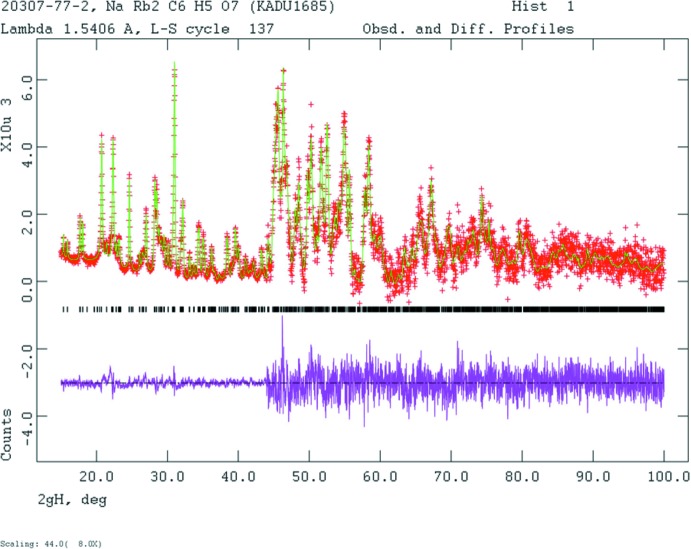
Rietveld plot for NaRb_2_C_6_H_5_O_7_. The red crosses represent the observed data points, and the green line is the calculated pattern. The magenta curve is the difference pattern, plotted at the same scale as the other patterns. The vertical scale has been multiplied by a factor of 8 for 2θ > 44.0°. The row of black tick marks indicates the reflection positions for this phase.

Crystal data, data collection and structure refinement (Fig. 8 ▸) details are summarized in Table 3 ▸. The diffraction patterns of both compounds were indexed using *N-TREOR* (Altomare *et al.*, 2013 ▸), and the cells were reduced using the tools in the PDF-4+ database (Fawcett *et al.*, 2017 ▸). The systematic absences in the the pattern of NaRb_2_C_6_H_5_O_7_(H_2_O)_2_ suggested the space groups *Pna2_1_* and *Pnam*. The unit-cell volume indicates that Z = 4, so *Pna2_1_* was chosen, and confirmed by successful solution and refinement of the structure.

The structure of NaRb_2_HC_6_H_5_O_7_ was solved using Monte Carlo simulated annealing techniques as implemented in *EXPO2014* (Altomare *et al.*, 2013 ▸). A citrate anion, a Na cation, and two Rb cations were used as fragments. The position of the active hydrogen atom H18 was deduced from the potential intra­molecular hydrogen-bonding pattern. Pseudovoigt profile coefficients were as parameterized in Thompson *et al.* (1987 ▸) and the asymmetry correction of Finger *et al.* (1994 ▸) was applied and microstrain broadening by Stephens (1999 ▸). The hydrogen atoms were included in fixed positions, which were re-calculated during the course of the refinement. The *U*_iso_ values of C2, C3, and C4 were constrained to be equal, and those of H7, H8, H9, and H10 were constrained to be 1.3 times that of these carbon atoms. The *U*_iso_ values of C1, C5, C6, and the oxygen atoms were constrained to be equal, and that of H18 was constrained to be 1.3 times this value. The *U*_iso_ values of Rb20 and Rb21 were constrained to be equal.

**Figure 9:**
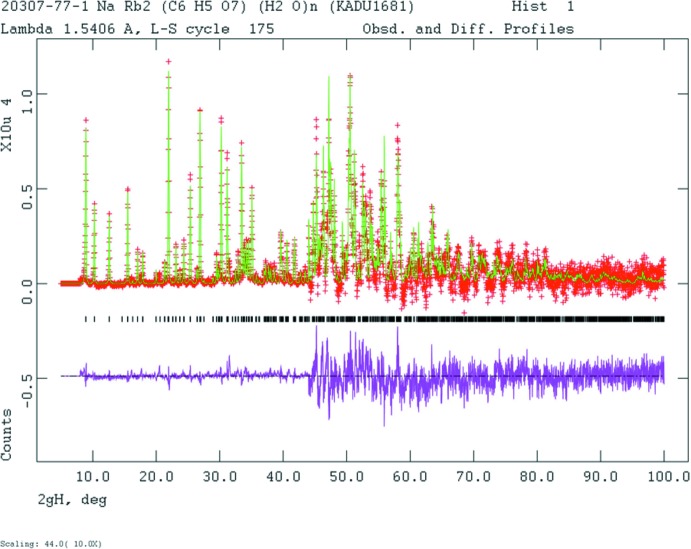
Rietveld plot for NaRb_2_C_6_H_5_O_7_(H_2_O)_2_. The red crosses represent the observed data points, and the green line is the calculated pattern. The magenta curve is the difference pattern, plotted at the same scale as the other patterns. The vertical scale has been multiplied by a factor of 10 for 2θ > 44.0°. The row of black tick marks indicates the reflection positions for this phase.

The structure of NaRb_2_C_6_H_5_O_7_(H_2_O)_2_ was solved using Monte Carlo simulated annealing techniques as implemented in *EXPO2014* (Altomare *et al.*, 2013 ▸). A citrate anion, a Na cation, two Rb cations, and three O atoms were used as fragments. In the best solution, one of the oxygen atoms was 1.30 Å from one of the Rb atoms, and was removed from the model. The positions of the active hydrogen atoms were deduced from potential hydrogen-bonding patterns. The same refinement strategy was used as for the anhydrous compound, and the *U*_iso_ values of the two water mol­ecule oxygen atoms were constrained to be equal. Comparison of the initial refined model to that from the DFT calculation revealed that the orientations of the carboxyl group C5/O13/O14 differed, so the Rietveld refinement (Fig. 9 ▸) was re-started from the DFT model.

Density functional geometry optimizations (fixed experimental unit cells) were carried out using *CRYSTAL14* (Dovesi *et al.*, 2014 ▸). The basis sets for the H, C, and O atoms were those of Gatti *et al.* (1994 ▸), the basis sets for Na was that of Dovesi *et al.* (1991 ▸), and the basis set for Rb was that of Sophia *et al.* (2014 ▸). The calculations were run on eight 2.1 GHz Xeon cores (each with 6 GB RAM) of a 304-core Dell Linux cluster at Illinois Institute of Technology, using 8 *k*-points and the B3LYP functional, and took approximately 5 and 29 h.

## Supplementary Material

Crystal structure: contains datablock(s) KADU1685_publ, kadu1685_DFT, KADU1681_publ, kadu1681_DFT. DOI: 10.1107/S2056989019003190/vn2141sup1.cif

Supporting information file. DOI: 10.1107/S2056989019003190/vn2141KADU1685_publsup2.cml

Supporting information file. DOI: 10.1107/S2056989019003190/vn2141KADU1681_publsup3.cml

CCDC references: 1901423, 1901422, 1901421, 1901420

Additional supporting information:  crystallographic information; 3D view; checkCIF report

## Figures and Tables

**Figure 2:**
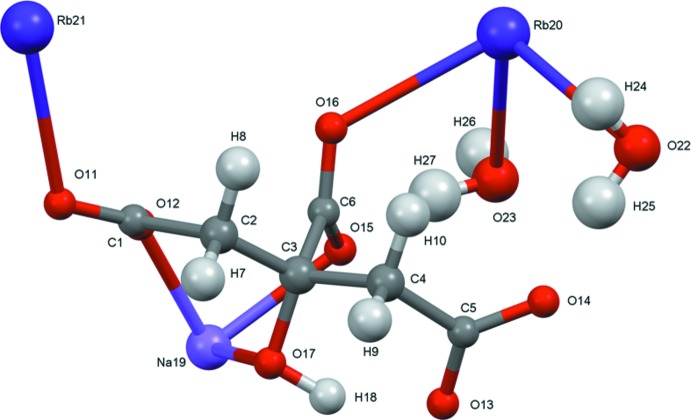
The asymmetric unit of NaRb_2_HC_6_H_5_O_7_(H_2_O)_2_, with the atom numbering and 50% probability spheroids.

**Table 1:** Hydrogen-bond geometry (Å, °, electrons, kcal mol^−1^) for [NaRb_2_(C_6_H_5_O_7_)]

*D*—H⋯*A*′	*D*—H	H⋯*A*	*D*⋯*A*	*D*—H⋯*A*	Mulliken overlap	H-bond energy
O17—H18⋯O11	0.996	1.662	2.585	152.3	0.072	14.7
C4—H10⋯O17^i^	1.088	2.451	3.515	165.5	0.017	

Symmetry code: (i) 1 + *x*, *y*, *z*.

**Table 2:** Hydrogen-bond geometry (Å, °, electrons, kcal mol^−1^) for [NaRb_2_(C_6_H_5_O_7_)(H_2_O)_2_]

*D*—H⋯*A*′	*D*—H	H⋯*A*	*D*⋯*A*	*D*—H⋯*A*	Mulliken overlap	H-bond energy
O23—H27⋯O15	0.986	1.755	2.721	165.6	0.064	13.8
O23—H26⋯O14^i^	0.974	1.934	2.833	152.2	0.041	11.1
O22—H25⋯O14^ii^	0.979	1.762	2.708	161.4	0.055	12.8
O22—H24⋯O13	0.980	1.779	2.718	159.0	0.053	12.6
O17—H18⋯O13	0.987	1.705	2.613	151.0	0.066	14.0
C4—H9⋯O13^ii^	1.096	2.402	3.374	147.0	0.016	

Symmetry code: (i) −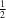 + *x*, 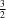 − *y*, *z*; (ii) *x*, *y*, −1 + *z*; (iii) 1 − *x*, 1 − *y*, 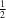 + *z*.

**Table 3:** Experimental details

	[NaRb_2_(C_6_H_5_O_7_)]	[NaRb_2_(C_6_H_5_O_7_)(H_2_O)_2_]
Crystal data		
*M*_r_	383.02	419.05
Crystal system, space group	Triclinic, *P* 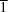	Orthorhombic, *P* *n* *a*2_1_
Temperature (K)	300	300
*a*, *b*, *c* (Å)	5.5917 (4), 7.8862 (5), 11.6133 (6)	12.1101 (3), 17.2422 (5), 5.73715 (18)
α, β, γ (°)	83.456 (4), 89.243 (5), 84.488 (4)	90, 90, 90
*V* (Å^3^)	506.42 (8)	1197.94 (8)
*Z*	2	4
Radiation type	Cu *K*α_1_, Cu *K*α_2_, λ = 1.540593, 1.544451 Å	*K*α_1_, *K*α_2_, λ = 1.540593, 1.544451 Å
Specimen shape, size (mm)	Flat sheet, 25 × 25	Flat sheet, 25 × 25

Data collection		
Diffractometer	Bruker D2 Phaser	Bruker D2 Phaser
Specimen mounting	Standard PMMA holder	Standard PMMA holder
Data collection mode	Reflection	Reflection
Scan method	Step	Step
2θ values (°)	2θ_min_ = 5.001 2θ_max_ = 100.007 2θ_step_ = 0.020	2θ_min_ = 5.001 2θ_max_ = 100.007 2θ_step_ = 0.020

Refinement		
*R* factors and goodness of fit	*R*_p_ = 0.023, *R*_wp_ = 0.029, *R*_exp_ = 0.022, *R*(*F* ^2^) = 0.06119, χ^2^ = 1.742	*R*_p_ = 0.035, *R*_wp_ = 0.047, *R*_exp_ = 0.023, *R*(*F* ^2^) = 0.21645, χ^2^ = 4.494
No. of parameters	75	67
No. of restraints	29	29
H-atom treatment	Only H-atom displacement parameters refined	Only H-atom displacement parameters refined

The same symmetry and lattice parameters were used for the DFT calculations as for each powder diffraction study. Computer programs: *Diffrac.Measurement* (Bruker, 2009 ▸), *PowDLL* (Kourkoumelis, 2013 ▸), *EXPO2014* (Altomare *et al.*), 2013 ▸), *GSAS* (Larson & Von Dreele, 2004 ▸), *Mercury* (Macrae *et al.*, 2008 ▸), *DIAMOND* (Crystal Impact, 2015 ▸) and *publCIF* (Westrip, 2010 ▸).

## supplementary crystallographic information

### Poly[µ-citrato-dirubidium(I)sodium(I)] (KADU1685_publ) Crystal data

**Table:**

[NaRb_2_(C_6_H_5_O_7_)]	γ = 84.488 (4)°
*M**_r_* = 383.02	*V* = 506.42 (8) Å^3^
Triclinic, *P*1	*Z* = 2
Hall symbol: -P 1	*D*_x_ = 2.512 Mg m^−^^3^
*a* = 5.5917 (4) Å	CuKα_1_, CuKα_2_ radiation, λ = 1.540593, 1.544451 Å
*b* = 7.8862 (5) Å	*T* = 300 K
*c* = 11.6133 (6) Å	pale yellow
α = 83.456 (4)°	flat_sheet, 25 × 25 mm
β = 89.243 (5)°	Specimen preparation: Prepared at 450 K

### Poly[µ-citrato-dirubidium(I)sodium(I)] (KADU1685_publ) Data collection

**Table:**

Bruker D2 Phaser diffractometer	Data collection mode: reflection
Radiation source: sealed Xray tube	Scan method: step
Ni filter monochromator	2θ_min_ = 5.001°, 2θ_max_ = 100.007°, 2θ_step_ = 0.020°
Specimen mounting: standard PMMA holder	

### Poly[µ-citrato-dirubidium(I)sodium(I)] (KADU1685_publ) Refinement

**Table:**

Least-squares matrix: full	Profile function: CW Profile function number 4 with 27 terms Pseudovoigt profile coefficients as parameterized in P. Thompson, D.E. Cox & J.B. Hastings (1987). J. Appl. Cryst.,20,79-83. Asymmetry correction of L.W. Finger, D.E. Cox & A. P. Jephcoat (1994). J. Appl. Cryst.,27,892-900. Microstrain broadening by P.W. Stephens, (1999). J. Appl. Cryst.,32,281-289. #1(GU) = 0.000 #2(GV) = 0.000 #3(GW) = 5.109 #4(GP) = 0.000 #5(LX) = 6.277 #6(ptec) = 0.00 #7(trns) = 1.30 #8(shft) = -2.9593 #9(sfec) = 0.00 #10(S/L) = 0.0005 #11(H/L) = 0.0097 #12(eta) = 0.9000 Peak tails are ignored where the intensity is below 0.0100 times the peak Aniso. broadening axis 0.0 0.0 1.0
*R*_p_ = 0.023	75 parameters
*R*_wp_ = 0.029	29 restraints
*R*_exp_ = 0.022	Only H-atom displacement parameters refined
*R*(*F*^2^) = 0.06119	Weighting scheme based on measured s.u.'s
4701 data points	(Δ/σ)_max_ = 0.01
Excluded region(s): The region from 5-15 degrees was excluded to minimize the effects of beam spillover and surface roughness.	Background function: GSAS Background function number 1 with 3 terms. Shifted Chebyshev function of 1st kind 1: 1719.95 2: -345.196 3: 91.9837

### Poly[µ-citrato-dirubidium(I)sodium(I)] (KADU1685_publ) Fractional atomic coordinates and isotropic or equivalent isotropic displacement parameters (Å^2^)

**Table:**

	*x*	*y*	*z*	*U*_iso_*/*U*_eq_	
C1	0.124 (2)	−0.2349 (14)	0.1360 (11)	0.010 (3)*	
C2	0.250 (3)	−0.0724 (15)	0.1222 (10)	0.023 (7)*	
C3	0.1816 (19)	0.0398 (11)	0.2201 (7)	0.023 (7)*	
C4	0.319 (3)	0.2010 (14)	0.2001 (10)	0.023 (7)*	
C5	0.286 (2)	0.3027 (17)	0.3028 (11)	0.010 (3)*	
C6	0.251 (2)	−0.0599 (17)	0.3397 (9)	0.010 (3)*	
H7	0.20437	−0.00565	0.04596	0.030 (9)*	
H8	0.42788	−0.10242	0.12401	0.030 (9)*	
H9	0.25594	0.27385	0.12877	0.030 (9)*	
H10	0.49370	0.16615	0.18985	0.030 (9)*	
O11	−0.099 (2)	−0.227 (2)	0.1578 (14)	0.010 (3)*	
O12	0.225 (3)	−0.3679 (14)	0.0961 (14)	0.010 (3)*	
O13	0.465 (3)	0.366 (2)	0.3415 (15)	0.010 (3)*	
O14	0.075 (3)	0.351 (2)	0.3358 (13)	0.010 (3)*	
O15	0.088 (3)	−0.118 (2)	0.4057 (11)	0.010 (3)*	
O16	0.457 (3)	−0.051 (2)	0.3819 (13)	0.010 (3)*	
O17	−0.070 (2)	0.0903 (16)	0.2172 (12)	0.010 (3)*	
H18	−0.12093	−0.00352	0.20357	0.013 (4)*	
Na19	−0.247 (3)	0.1397 (17)	0.4081 (11)	0.010 (5)*	
Rb20	0.7394 (9)	0.4509 (7)	0.1312 (3)	0.0224 (13)*	
Rb21	−0.2414 (10)	−0.3662 (5)	0.4096 (3)	0.0224 (13)*	

### Poly[µ-citrato-dirubidium(I)sodium(I)] (KADU1685_publ) Geometric parameters (Å, º)

**Table:**

C1—C2	1.5108 (13)	O14—Rb21^vi^	3.130 (17)
C1—O11	1.269 (4)	O15—C6	1.269 (4)
C1—O12	1.273 (4)	O15—Na19	2.63 (2)
C2—C1	1.5108 (13)	O15—Na19^vi^	2.33 (2)
C2—C3	1.5411 (13)	O15—Rb21	2.810 (17)
C3—C2	1.5411 (13)	O16—C6	1.270 (4)
C3—C4	1.5405 (13)	O16—Na19^iv^	2.38 (2)
C3—C6	1.5507 (13)	O16—Na19^vi^	2.74 (2)
C3—O17	1.423 (4)	O16—Rb21^iv^	2.858 (16)
C4—C3	1.5405 (13)	O17—C3	1.423 (4)
C4—C5	1.5100 (13)	O17—Na19	2.473 (18)
C5—C4	1.5100 (13)	O17—Rb20^vii^	3.003 (13)
C5—O13	1.270 (4)	Na19—O13^vii^	2.35 (2)
C5—O14	1.269 (4)	Na19—O14	2.637 (19)
C6—C3	1.5507 (13)	Na19—O15	2.63 (2)
C6—O15	1.269 (4)	Na19—O15^vi^	2.33 (2)
C6—O16	1.270 (4)	Na19—O16^vii^	2.38 (2)
O11—C1	1.269 (4)	Na19—O16^vi^	2.74 (2)
O11—Rb20^i^	2.823 (13)	Na19—O17	2.473 (18)
O11—Rb21	3.124 (16)	Rb20—O11^v^	2.823 (13)
O12—C1	1.273 (4)	Rb20—O12^viii^	3.098 (16)
O12—Rb20^i^	3.187 (16)	Rb20—O12^v^	3.187 (16)
O12—Rb20^ii^	3.098 (16)	Rb20—O12^iii^	2.791 (15)
O12—Rb20^iii^	2.791 (15)	Rb20—O13	2.914 (19)
O13—C5	1.270 (4)	Rb20—O14^iv^	3.034 (19)
O13—Na19^iv^	2.35 (2)	Rb20—O17^iv^	3.003 (13)
O13—Rb20	2.914 (19)	Rb21—O11	3.124 (16)
O13—Rb21^v^	2.978 (12)	Rb21—O13^i^	2.978 (12)
O13—Rb21^vi^	3.135 (19)	Rb21—O13^vi^	3.135 (19)
O14—C5	1.269 (4)	Rb21—O14^ii^	2.912 (14)
O14—Na19	2.637 (19)	Rb21—O14^vi^	3.130 (17)
O14—Rb20^vii^	3.034 (19)	Rb21—O15	2.810 (17)
O14—Rb21^viii^	2.912 (14)	Rb21—O16^vii^	2.858 (16)
			
C2—C1—O11	119.8 (5)	O13^vii^—Na19—O14	85.8 (5)
C2—C1—O12	119.0 (4)	O13^vii^—Na19—O15	160.3 (8)
O11—C1—O12	118.7 (3)	O13^vii^—Na19—O15^vi^	121.1 (8)
O11—C1—Rb20^i^	51.1 (6)	O13^vii^—Na19—O16^vii^	87.2 (8)
O12—C1—Rb20^i^	67.9 (5)	O13^vii^—Na19—O16^vi^	97.3 (8)
C1—C2—C3	111.6 (4)	O13^vii^—Na19—O17	97.2 (7)
C2—C3—C4	108.2 (3)	O14—Na19—O15	89.0 (8)
C2—C3—C6	110.4 (4)	O14—Na19—O15^vi^	89.2 (7)
C2—C3—O17	109.9 (4)	O14—Na19—O16^vii^	154.3 (7)
C4—C3—C6	109.6 (3)	O14—Na19—O16^vi^	134.2 (7)
C4—C3—O17	109.1 (4)	O14—Na19—O17	66.1 (6)
C6—C3—O17	109.7 (3)	O15—Na19—O15^vi^	77.7 (8)
C3—C4—C5	110.2 (4)	O15—Na19—O16^vii^	89.3 (6)
C4—C5—O13	119.4 (4)	O15—Na19—O16^vi^	99.9 (6)
C4—C5—O14	119.7 (3)	O15—Na19—O17	63.5 (5)
O13—C5—O14	119.6 (5)	O15^vi^—Na19—O16^vii^	115.4 (7)
O13—C5—Rb20	56.5 (8)	O15^vi^—Na19—O16^vi^	50.3 (3)
O13—C5—Rb21^vi^	65.7 (8)	O15^vi^—Na19—O17	133.2 (9)
O14—C5—Rb20	140.2 (13)	O16^vii^—Na19—O16^vi^	71.3 (7)
O14—C5—Rb21^vi^	65.4 (8)	O16^vii^—Na19—O17	90.3 (7)
C1—O11—Rb20^i^	108.4 (7)	O16^vi^—Na19—O17	155.9 (7)
C1—O11—Rb21	116.0 (11)	O11^v^—Rb20—O12^viii^	88.4 (4)
Rb20^i^—O11—Rb21	76.6 (4)	O11^v^—Rb20—O12^v^	42.1 (2)
C1—O12—Rb20^i^	90.4 (5)	O11^v^—Rb20—O12^iii^	113.3 (4)
C1—O12—Rb20^ii^	130.7 (9)	O11^v^—Rb20—O13	104.5 (4)
C1—O12—Rb20^iii^	130.3 (12)	O11^v^—Rb20—O14^iv^	79.8 (5)
Rb20^i^—O12—Rb20^ii^	125.7 (4)	O11^v^—Rb20—O17^iv^	132.4 (4)
Rb20^i^—O12—Rb20^iii^	90.3 (4)	O12^viii^—Rb20—O12^v^	125.7 (4)
Rb20^ii^—O12—Rb20^iii^	86.5 (4)	O12^viii^—Rb20—O12^iii^	93.5 (4)
C5—O13—Na19^iv^	108.6 (13)	O12^viii^—Rb20—O13	72.1 (5)
C5—O13—Rb20	102.2 (10)	O12^viii^—Rb20—O14^iv^	135.5 (4)
C5—O13—Rb21^v^	158.1 (13)	O12^viii^—Rb20—O17^iv^	133.1 (4)
C5—O13—Rb21^vi^	92.7 (9)	O12^v^—Rb20—O12^iii^	89.7 (4)
Na19^iv^—O13—Rb20	92.1 (7)	O12^v^—Rb20—O13	130.1 (4)
Na19^iv^—O13—Rb21^v^	93.3 (5)	O12^v^—Rb20—O14^iv^	68.4 (5)
Na19^iv^—O13—Rb21^vi^	90.8 (7)	O12^v^—Rb20—O17^iv^	101.1 (4)
Rb20—O13—Rb21^v^	77.6 (4)	O12^iii^—Rb20—O13	139.1 (4)
Rb20—O13—Rb21^vi^	163.0 (4)	O12^iii^—Rb20—O14^iv^	130.6 (5)
Rb21^v^—O13—Rb21^vi^	85.5 (4)	O12^iii^—Rb20—O17^iv^	89.5 (4)
C5—O14—Na19	123.9 (14)	O13—Rb20—O14^iv^	69.8 (3)
C5—O14—Rb20^vii^	111.3 (9)	O13—Rb20—O17^iv^	75.4 (5)
C5—O14—Rb21^viii^	145.9 (12)	O14^iv^—Rb20—O17^iv^	55.0 (4)
C5—O14—Rb21^vi^	92.9 (8)	O11—Rb21—O13^i^	96.0 (5)
Na19—O14—Rb20^vii^	84.2 (5)	O11—Rb21—O13^vi^	158.9 (5)
Na19—O14—Rb21^viii^	89.2 (5)	O11—Rb21—O14^ii^	77.0 (5)
Na19—O14—Rb21^vi^	91.2 (6)	O11—Rb21—O14^vi^	138.7 (4)
Rb20^vii^—O14—Rb21^viii^	76.8 (4)	O11—Rb21—O15	67.5 (3)
Rb20^vii^—O14—Rb21^vi^	153.5 (5)	O11—Rb21—O16^vii^	80.2 (5)
Rb21^viii^—O14—Rb21^vi^	77.1 (3)	O13^i^—Rb21—O13^vi^	94.5 (4)
C6—O15—Na19	105.0 (9)	O13^i^—Rb21—O14^ii^	70.6 (3)
C6—O15—Na19^vi^	104.8 (10)	O13^i^—Rb21—O14^vi^	123.3 (6)
C6—O15—Rb21	138.4 (12)	O13^i^—Rb21—O15	162.7 (5)
Na19—O15—Na19^vi^	102.3 (8)	O13^i^—Rb21—O16^vii^	106.4 (5)
Na19—O15—Rb21	94.2 (6)	O13^vi^—Rb21—O14^ii^	123.9 (6)
Na19^vi^—O15—Rb21	106.6 (5)	O13^vi^—Rb21—O14^vi^	41.01 (19)
C6—O16—Na19^iv^	143.9 (11)	O13^vi^—Rb21—O15	102.7 (3)
C6—O16—Na19^vi^	85.3 (8)	O13^vi^—Rb21—O16^vii^	79.4 (5)
C6—O16—Rb21^iv^	115.0 (12)	O14^ii^—Rb21—O14^vi^	102.9 (3)
Na19^iv^—O16—Na19^vi^	108.7 (7)	O14^ii^—Rb21—O15	99.4 (5)
Na19^iv^—O16—Rb21^iv^	98.5 (6)	O14^ii^—Rb21—O16^vii^	156.4 (4)
Na19^vi^—O16—Rb21^iv^	89.6 (5)	O14^vi^—Rb21—O15	71.9 (4)
C3—O17—Na19	114.2 (7)	O14^vi^—Rb21—O16^vii^	98.0 (4)
C3—O17—Rb20^vii^	121.1 (6)	O15—Rb21—O16^vii^	76.9 (3)
Na19—O17—Rb20^vii^	87.7 (5)		

Symmetry codes: (i) *x*−1, *y*−1, *z*; (ii) *x*, *y*−1, *z*; (iii) −*x*+1, −*y*, −*z*; (iv) *x*+1, *y*, *z*; (v) *x*+1, *y*+1, *z*; (vi) −*x*, −*y*, −*z*+1; (vii) *x*−1, *y*, *z*; (viii) *x*, *y*+1, *z*.

### (kadu1685_DFT) Crystal data

**Table:**

C6H5NaO7Rb_2_	α = 83.4560°
*M**_r_* = 383.02	β = 89.2430°
Triclinic, *P*1	γ = 84.4880°
*a* = 5.5917 Å	*V* = 506.42 Å^3^
*b* = 7.8862 Å	*Z* = 2
*c* = 11.6133 Å	

### (kadu1685_DFT) Data collection

**Table:**

*h* = →	*l* = →
*k* = →	

### (kadu1685_DFT) Fractional atomic coordinates and isotropic or equivalent isotropic displacement parameters (Å^2^)

**Table:**

	*x*	*y*	*z*	*U*_iso_*/*U*_eq_	
C1	0.12954	−0.24091	0.13258	0.01020*	
C2	0.26324	−0.07916	0.13201	0.02320*	
C3	0.17418	0.04028	0.22411	0.02320*	
C4	0.29971	0.20638	0.20696	0.02320*	
C5	0.27264	0.31378	0.31043	0.01020*	
C6	0.24131	−0.05121	0.34701	0.01020*	
H7	0.23583	−0.00707	0.04568	0.03000*	
H8	0.45556	−0.11740	0.14158	0.03000*	
H9	0.22830	0.28512	0.12888	0.03000*	
H10	0.48942	0.17037	0.19211	0.03000*	
O11	−0.09135	−0.22959	0.16288	0.01020*	
O12	0.24168	−0.37299	0.09935	0.01020*	
O13	0.46259	0.36227	0.34992	0.01020*	
O14	0.06361	0.34542	0.35028	0.01020*	
O15	0.07766	−0.11340	0.40960	0.01020*	
O16	0.45856	−0.05647	0.37654	0.01020*	
O17	−0.07963	0.08108	0.21210	0.01020*	
H18	−0.13612	−0.03134	0.19953	0.01300*	
Na19	−0.23798	0.12272	0.40262	0.01000*	
Rb20	0.74402	0.44949	0.13375	0.02240*	
Rb21	−0.24917	−0.36450	0.40909	0.02240*	

### (kadu1685_DFT) Bond lengths (Å)

**Table:**

C1—C2	1.539	O14—Na19	2.568
C1—O11	1.277	O14—Rb20^vii^	3.091
C1—O12	1.260	O14—Rb21^v^	3.018
C2—C3	1.551	O14—Rb21^viii^	2.884
C2—H7	1.100	O15—Na19^v^	2.359
C2—H8	1.092	O15—Rb21	2.821
C3—C4	1.537	O16—Na19^iv^	2.354
C3—C6	1.558	O16—Rb21^iv^	2.785
C3—O17	1.430	O17—H18	0.996
C4—C5	1.545	O17—Na19	2.418
C4—H9	1.096	O17—Rb20^vii^	3.019
C4—H10	1.088	Na19—O15^v^	2.359
C5—O13	1.271	Na19—O13^vii^	2.429
C5—O14	1.264	Na19—O16^vii^	2.354
C6—O15	1.262	Rb20—O12^viii^	3.026
C6—O16	1.262	Rb20—O14^iv^	3.091
O11—Rb20^i^	2.832	Rb20—O17^iv^	3.019
O11—Rb21	3.083	Rb20—O11^vi^	2.832
O12—Rb20^ii^	3.026	Rb20—O12^vi^	3.233
O12—Rb20^i^	3.233	Rb20—O12^iii^	2.838
O12—Rb20^iii^	2.838	Rb21—O16^vii^	2.785
O13—Na19^iv^	2.429	Rb21—O13^v^	3.029
O13—Rb20	2.994	Rb21—O14^v^	3.018
O13—Rb21^v^	3.029	Rb21—O13^i^	2.957
O13—Rb21^vi^	2.957	Rb21—O14^ii^	2.884

Symmetry codes: (i) *x*−1, *y*−1, *z*; (ii) *x*, *y*−1, *z*; (iii) −*x*+1, −*y*, −*z*; (iv) *x*+1, *y*, *z*; (v) −*x*, −*y*, −*z*+1; (vi) *x*+1, *y*+1, *z*; (vii) *x*−1, *y*, *z*; (viii) *x*, *y*+1, *z*.

### (kadu1685_DFT) Hydrogen-bond geometry (Å, º)

**Table:**

*D*—H···*A*	*D*—H	H···*A*	*D*···*A*	*D*—H···*A*
O17—H18···O11	0.996	1.662	2.585	152.3
C4—H10···O17	1.088	2.451	3.515	165.5

### Poly[diaqua(µ-citrato)dirubidium(I)sodium(I)] (KADU1681_publ) Crystal data

**Table:**

[NaRb_2_(C_6_H_5_O_7_)(H_2_O)_2_]	*V* = 1197.94 (8) Å^3^
*M**_r_* = 419.05	*Z* = 4
Orthorhombic, *P**n**a*2_1_	*D*_x_ = 2.342 Mg m^−^^3^
Hall symbol: P 2c -2n	Kα_1_, Kα_2_ radiation, λ = 1.540593, 1.544451 Å
*a* = 12.1101 (3) Å	*T* = 300 K
*b* = 17.2422 (5) Å	flat_sheet, 25 × 25 mm
*c* = 5.73715 (18) Å	Specimen preparation: Prepared at 450 K

### Poly[diaqua(µ-citrato)dirubidium(I)sodium(I)] (KADU1681_publ) Data collection

**Table:**

Bruker D2 Phaser diffractometer	Data collection mode: reflection
Radiation source: sealed X-ray tube	Scan method: step
Ni filter monochromator	2θ_min_ = 5.001°, 2θ_max_ = 100.007°, 2θ_step_ = 0.020°
Specimen mounting: standard PMMA holder	

### Poly[diaqua(µ-citrato)dirubidium(I)sodium(I)] (KADU1681_publ) Refinement

**Table:**

Least-squares matrix: full	67 parameters
*R*_p_ = 0.035	29 restraints
*R*_wp_ = 0.047	Only H-atom displacement parameters refined
*R*_exp_ = 0.023	Weighting scheme based on measured s.u.'s
*R*(*F*^2^) = 0.21645	(Δ/σ)_max_ = 0.04
4701 data points	Background function: GSAS Background function number 1 with 3 terms. Shifted Chebyshev function of 1st kind 1: 1693.18 2: -250.425 3: 22.2802
Profile function: CW Profile function number 4 with 18 terms Pseudovoigt profile coefficients as parameterized in P. Thompson, D.E. Cox & J.B. Hastings (1987). J. Appl. Cryst.,20,79-83. Asymmetry correction of L.W. Finger, D.E. Cox & A. P. Jephcoat (1994). J. Appl. Cryst.,27,892-900. Microstrain broadening by P.W. Stephens, (1999). J. Appl. Cryst.,32,281-289. #1(GU) = 0.000 #2(GV) = 0.000 #3(GW) = 5.109 #4(GP) = 0.000 #5(LX) = 3.634 #6(ptec) = 0.00 #7(trns) = 1.30 #8(shft) = -4.0778 #9(sfec) = 0.00 #10(S/L) = 0.0295 #11(H/L) = 0.0097 #12(eta) = 0.9000 #13(S400 ) = 3.3E-04 #14(S040 ) = 2.5E-05 #15(S004 ) = 0.0E+00 #16(S220 ) = 6.5E-03 #17(S202 ) = 9.2E-04 #18(S022 ) = 3.0E-03 Peak tails are ignored where the intensity is below 0.0100 times the peak Aniso. broadening axis 0.0 0.0 1.0	

### Poly[diaqua(µ-citrato)dirubidium(I)sodium(I)] (KADU1681_publ) Fractional atomic coordinates and isotropic or equivalent isotropic displacement parameters (Å^2^)

**Table:**

	*x*	*y*	*z*	*U*_iso_*/*U*_eq_	
C1	0.1222 (10)	0.4396 (8)	0.41415	0.024 (4)*	
C2	0.2395 (12)	0.4503 (9)	0.498 (5)	0.034 (12)*	
C3	0.2929 (9)	0.5252 (5)	0.405 (5)	0.034 (12)*	
C4	0.4049 (12)	0.5361 (8)	0.528 (6)	0.034 (12)*	
C5	0.4633 (12)	0.6091 (6)	0.450 (6)	0.024 (4)*	
C6	0.2178 (17)	0.5957 (9)	0.460 (6)	0.024 (4)*	
H7	0.28466	0.40490	0.44629	0.045 (15)*	
H8	0.24038	0.45278	0.67213	0.045 (15)*	
H9	0.45297	0.49037	0.49350	0.045 (15)*	
H10	0.39304	0.53952	0.70018	0.045 (15)*	
O11	0.0717 (10)	0.3782 (9)	0.471 (7)	0.024 (4)*	
O12	0.0665 (13)	0.4969 (9)	0.337 (6)	0.024 (4)*	
O13	0.5481 (13)	0.6042 (9)	0.322 (7)	0.024 (4)*	
O14	0.4444 (16)	0.6727 (8)	0.552 (7)	0.024 (4)*	
O15	0.196 (2)	0.6441 (11)	0.301 (7)	0.024 (4)*	
O16	0.195 (2)	0.6118 (13)	0.670 (6)	0.024 (4)*	
O17	0.3087 (15)	0.5187 (14)	0.159 (5)	0.024 (4)*	
H18	0.34428	0.55892	0.13326	0.031 (5)*	
Na19	0.1149 (15)	0.5624 (9)	−0.072 (8)	0.048 (8)*	
Rb20	0.3036 (3)	0.7303 (2)	0.995 (6)	0.0662 (17)*	
Rb21	0.0285 (3)	0.3392 (3)	0.968 (5)	0.0662 (17)*	
O22	0.5625 (14)	0.7125 (12)	−0.060 (9)	0.05*	
O23	0.2385 (16)	0.7991 (12)	0.489 (12)	0.05*	
H24	0.52628	0.68621	0.03931	0.065*	
H25	0.54984	0.69167	−0.19160	0.065*	
H26	0.17964	0.82566	0.50138	0.065*	
H27	0.21772	0.75215	0.47697	0.065*	

### Poly[diaqua(µ-citrato)dirubidium(I)sodium(I)] (KADU1681_publ) Geometric parameters (Å, º)

**Table:**

C1—C2	1.511 (2)	O16—C6	1.267 (6)
C1—O11	1.265 (6)	O16—Na19^vi^	1.97 (4)
C1—O12	1.275 (6)	O16—Rb20	3.06 (3)
C2—C1	1.511 (2)	O16—Rb21^iv^	3.07 (3)
C2—C3	1.541 (2)	O17—C3	1.426 (6)
C3—C2	1.541 (2)	O17—Na19	2.80 (3)
C3—C4	1.541 (2)	O17—Rb20^iii^	3.77 (2)
C3—C6	1.550 (2)	Na19—O11^iv^	2.49 (2)
C3—O17	1.426 (6)	Na19—O12	2.67 (4)
C4—C3	1.541 (2)	Na19—O12^iv^	2.48 (2)
C4—C5	1.512 (2)	Na19—O15	2.74 (4)
C5—C4	1.512 (2)	Na19—O16^iii^	1.97 (4)
C5—O13	1.264 (6)	Na19—O17	2.80 (3)
C5—O14	1.264 (6)	Rb20—O11^vii^	2.967 (14)
C6—C3	1.550 (2)	Rb20—O14	3.22 (2)
C6—O15	1.266 (6)	Rb20—O14^vi^	3.76 (3)
C6—O16	1.267 (6)	Rb20—O15^vi^	2.65 (3)
O11—C1	1.265 (6)	Rb20—O16	3.06 (3)
O11—Na19^i^	2.49 (2)	Rb20—O17^vi^	3.77 (2)
O11—Rb20^ii^	2.967 (14)	Rb20—O22^vi^	3.165 (18)
O11—Rb21^iii^	3.01 (4)	Rb20—O22^viii^	3.099 (18)
O11—Rb21	2.97 (4)	Rb20—O23	3.24 (7)
O12—C1	1.275 (6)	Rb20—O23^vi^	3.17 (7)
O12—Na19	2.67 (4)	Rb21—O11	2.97 (4)
O12—Na19^i^	2.48 (2)	Rb21—O11^vi^	3.01 (4)
O12—Rb21^iii^	3.48 (2)	Rb21—O12^vi^	3.48 (2)
O12—Rb21^iv^	3.142 (17)	Rb21—O12^i^	3.142 (17)
O13—C5	1.264 (6)	Rb21—O14^ix^	2.929 (15)
O13—O14	2.171 (10)	Rb21—O15^i^	2.89 (3)
O14—C5	1.264 (6)	Rb21—O16^i^	3.07 (3)
O14—O13	2.171 (10)	Rb21—O22^x^	3.65 (4)
O14—Rb20^iii^	3.76 (3)	Rb21—O23^ix^	2.91 (2)
O14—Rb20	3.22 (2)	O22—Rb20^iii^	3.165 (18)
O14—Rb21^v^	2.929 (15)	O22—Rb20^xi^	3.099 (18)
O15—C6	1.266 (6)	O22—Rb21^xii^	3.65 (4)
O15—Na19	2.74 (4)	O23—Rb20^iii^	3.17 (7)
O15—Rb20^iii^	2.65 (3)	O23—Rb20	3.24 (7)
O15—Rb21^iv^	2.89 (3)	O23—Rb21^v^	2.91 (2)
			
C2—C1—O11	118.3 (6)	O12^iv^—Na19—O15	133.7 (13)
C2—C1—O12	120.8 (6)	O12^iv^—Na19—O16^iii^	117.4 (19)
O11—C1—O12	118.8 (6)	O15—Na19—O16^iii^	100.9 (9)
C1—C2—C3	112.7 (5)	O11^vii^—Rb20—O14	87.7 (7)
C2—C3—C4	108.2 (5)	O11^vii^—Rb20—O15^vi^	140.0 (11)
C2—C3—C6	109.9 (5)	O11^vii^—Rb20—O16	139.7 (11)
C2—C3—O17	109.6 (6)	O11^vii^—Rb20—O22^vi^	64.8 (5)
C4—C3—C6	109.1 (5)	O11^vii^—Rb20—O22^viii^	101.6 (4)
C4—C3—O17	110.1 (6)	O11^vii^—Rb20—O23	76.5 (9)
C6—C3—O17	110.0 (5)	O11^vii^—Rb20—O23^vi^	81.1 (8)
C3—C4—C5	112.2 (5)	O14—Rb20—O15^vi^	127.8 (6)
C4—C5—O13	119.7 (6)	O14—Rb20—O16	62.6 (6)
C4—C5—O14	120.0 (6)	O14—Rb20—O22^vi^	50.7 (9)
O13—C5—O14	118.3 (5)	O14—Rb20—O22^viii^	121.2 (11)
C3—C6—O15	119.7 (6)	O14—Rb20—O23	62.2 (7)
C3—C6—O16	119.6 (6)	O14—Rb20—O23^vi^	162.3 (6)
O15—C6—O16	119.6 (6)	O15^vi^—Rb20—O22^vi^	120.1 (9)
C1—O11—Na19^i^	94.0 (9)	O15^vi^—Rb20—O22^viii^	77.3 (8)
C1—O11—Rb20^ii^	119.0 (11)	O15^vi^—Rb20—O23	132.9 (7)
C1—O11—Rb21^iii^	91.3 (19)	O15^vi^—Rb20—O23^vi^	59.7 (7)
C1—O11—Rb21	122 (2)	O16—Rb20—O22^vi^	107.4 (7)
Na19^i^—O11—Rb20^ii^	145.0 (7)	O16—Rb20—O22^viii^	75.3 (8)
Na19^i^—O11—Rb21^iii^	80.7 (12)	O16—Rb20—O23	66.0 (7)
Na19^i^—O11—Rb21	91.7 (12)	O16—Rb20—O23^vi^	133.4 (6)
Rb20^ii^—O11—Rb21^iii^	86.6 (9)	O22^vi^—Rb20—O22^viii^	162.5 (13)
Rb20^ii^—O11—Rb21	81.4 (7)	O22^vi^—Rb20—O23	100.8 (11)
Rb21^iii^—O11—Rb21	146.9 (5)	O22^vi^—Rb20—O23^vi^	111.8 (11)
C1—O12—Na19	121.0 (17)	O22^viii^—Rb20—O23	64.0 (10)
C1—O12—Na19^i^	94.4 (9)	O22^viii^—Rb20—O23^vi^	74.8 (10)
C1—O12—Rb21^iv^	144.0 (17)	O23—Rb20—O23^vi^	127.2 (6)
Na19—O12—Na19^i^	123.6 (13)	O11—Rb21—O11^vi^	146.9 (5)
Na19—O12—Rb21^iv^	84.8 (6)	O11—Rb21—O12^i^	68.4 (5)
Na19^i^—O12—Rb21^iv^	89.8 (6)	O11—Rb21—O14^ix^	111.2 (6)
C5—O14—Rb20	137.0 (10)	O11—Rb21—O15^i^	79.9 (7)
C5—O14—Rb21^v^	138.7 (12)	O11—Rb21—O16^i^	117.1 (6)
Rb20—O14—Rb21^v^	83.6 (4)	O11—Rb21—O23^ix^	85.6 (14)
C6—O15—Na19	107.4 (16)	O11^vi^—Rb21—O12^i^	95.2 (4)
C6—O15—Rb20^iii^	138 (2)	O11^vi^—Rb21—O14^ix^	92.3 (6)
C6—O15—Rb21^iv^	91.6 (15)	O11^vi^—Rb21—O15^i^	117.3 (6)
Na19—O15—Rb20^iii^	87.0 (10)	O11^vi^—Rb21—O16^i^	74.3 (5)
Na19—O15—Rb21^iv^	88.6 (9)	O11^vi^—Rb21—O23^ix^	81.1 (14)
Rb20^iii^—O15—Rb21^iv^	128.9 (5)	O12^i^—Rb21—O14^ix^	164.1 (5)
C6—O16—Na19^vi^	137 (2)	O12^i^—Rb21—O15^i^	59.2 (6)
C6—O16—Rb20	129 (2)	O12^i^—Rb21—O16^i^	61.2 (5)
C6—O16—Rb21^iv^	83.8 (15)	O12^i^—Rb21—O23^ix^	125.4 (6)
Na19^vi^—O16—Rb20	92.4 (12)	O14^ix^—Rb21—O15^i^	104.9 (6)
Na19^vi^—O16—Rb21^iv^	88.1 (12)	O14^ix^—Rb21—O16^i^	107.8 (6)
Rb20—O16—Rb21^iv^	115.2 (5)	O14^ix^—Rb21—O23^ix^	69.6 (5)
O11^iv^—Na19—O12	83.5 (11)	O15^i^—Rb21—O16^i^	43.0 (3)
O11^iv^—Na19—O12^iv^	52.2 (4)	O15^i^—Rb21—O23^ix^	161.4 (14)
O11^iv^—Na19—O15	92.0 (12)	O16^i^—Rb21—O23^ix^	155.2 (15)
O11^iv^—Na19—O16^iii^	110.2 (17)	Rb20^iii^—O22—Rb20^xi^	153.1 (9)
O12—Na19—O12^iv^	79.4 (8)	Rb20^iii^—O23—Rb20	127.2 (6)
O12—Na19—O15	67.0 (11)	Rb20^iii^—O23—Rb21^v^	79.1 (12)
O12—Na19—O16^iii^	162.5 (17)	Rb20—O23—Rb21^v^	83.6 (12)

Symmetry codes: (i) −*x*, −*y*+1, *z*+1/2; (ii) −*x*+1/2, *y*−1/2, *z*−1/2; (iii) *x*, *y*, *z*−1; (iv) −*x*, −*y*+1, *z*−1/2; (v) −*x*+1/2, *y*+1/2, *z*−1/2; (vi) *x*, *y*, *z*+1; (vii) −*x*+1/2, *y*+1/2, *z*+1/2; (viii) *x*−1/2, −*y*+3/2, *z*+1; (ix) −*x*+1/2, *y*−1/2, *z*+1/2; (x) −*x*+1/2, *y*−1/2, *z*+3/2; (xi) *x*+1/2, −*y*+3/2, *z*−1; (xii) −*x*+1/2, *y*+1/2, *z*−3/2.

### (kadu1681_DFT) Crystal data

**Table:**

C_6_H_9_NaO_9_Rb_2_	*b* = 17.2422 Å
*M**_r_* = 419.05	*c* = 5.7371 Å
Orthorhombic, *P**n**a*2_1_	*V* = 1197.94 Å^3^
*a* = 12.1101 Å	*Z* = 4

### (kadu1681_DFT) Data collection

**Table:**

*h* = →	*l* = →
*k* = →	

### (kadu1681_DFT) Fractional atomic coordinates and isotropic or equivalent isotropic displacement parameters (Å^2^)

**Table:**

	*x*	*y*	*z*	*U*_iso_*/*U*_eq_	
C1	0.11349	0.44294	0.51714	0.02400*	
C2	0.23597	0.45666	0.57421	0.03400*	
C3	0.28367	0.52794	0.45370	0.03400*	
C4	0.40500	0.54379	0.52712	0.03400*	
C5	0.45823	0.61018	0.38987	0.02400*	
C6	0.21792	0.60115	0.52617	0.02400*	
H7	0.28454	0.40572	0.52464	0.04500*	
H8	0.24316	0.46248	0.76364	0.04500*	
H9	0.45422	0.49133	0.49852	0.04500*	
H10	0.40815	0.55714	0.71290	0.04500*	
O11	0.07326	0.37761	0.57270	0.02400*	
O12	0.05725	0.49630	0.42197	0.02400*	
O13	0.44070	0.61196	0.17085	0.02400*	
O14	0.51674	0.65996	0.49354	0.02400*	
O15	0.19321	0.64945	0.36630	0.02400*	
O16	0.19781	0.61091	0.73943	0.02400*	
O17	0.27723	0.51384	0.20845	0.02400*	
H18	0.33394	0.54823	0.14074	0.03100*	
Na19	0.10971	0.56678	0.07196	0.04800*	
Rb20	0.28861	0.74043	0.99974	0.06620*	
Rb21	0.02821	0.33790	1.05852	0.06620*	
O22	0.55613	0.71905	−0.07701	0.05000*	
O23	0.24192	0.79591	0.51019	0.05000*	
H24	0.52483	0.68516	0.04328	0.06500*	
H25	0.54732	0.68753	−0.21777	0.06500*	
H26	0.17541	0.82757	0.50229	0.06500*	
H27	0.21390	0.74353	0.47482	0.06500*	

### (kadu1681_DFT) Bond lengths (Å)

**Table:**

C1—C2	1.537	C4—H10	1.091
C1—O11	1.268	C5—O13	1.275
C1—O12	1.268	C5—O14	1.262
C2—C3	1.524	C6—O15	1.275
C2—H7	1.095	C6—O16	1.259
C2—H8	1.095	O17—H18	0.987
C3—C4	1.553	O22—H24	0.980
C3—C6	1.549	O22—H25	0.979
C3—O17	1.430	O23—H26	0.974
C4—C5	1.532	O23—H27	0.986
C4—H9	1.096		

### (kadu1681_DFT) Hydrogen-bond geometry (Å, º)

**Table:**

*D*—H···*A*	*D*—H	H···*A*	*D*···*A*	*D*—H···*A*
O23—H27···O15	0.986	1.755	2.721	165.6
O23—H26···O14	0.974	1.934	2.833	152.2
O22—H25···O14	0.979	1.762	2.708	161.4
O22—H24···O13	0.980	1.779	2.718	159.0
O17—H18···O13	0.987	1.705	2.613	151.0
C4—H9···O13	1.096	2.402	3.374	147.0
